# Effects of Covid-19 Lockdown on Mental Health and Sleep Disturbances in Italy

**DOI:** 10.3390/ijerph17134779

**Published:** 2020-07-02

**Authors:** Maria Rosaria Gualano, Giuseppina Lo Moro, Gianluca Voglino, Fabrizio Bert, Roberta Siliquini

**Affiliations:** 1 Department of Public Health Sciences and Paediatrics, University of Torino, 10124 Torino, Italy; mariarosaria.gualano@unito.it (M.R.G.); giuseppina.lomoro@unito.it (G.L.M.); fabrizio.bert@unito.it (F.B.); roberta.siliquini@unito.it (R.S.); 2 Azienda Ospedaliero-Universitaria, City of Health and Science of Turin, 10126 Torino, Italy

**Keywords:** depression, anxiety, patient health questionnaire, sleep wake disorders, quarantine

## Abstract

Italy was the first European country that entered a nationwide lockdown during the COVID-19 pandemic. Since quarantine can impact on mental health, this study aimed to estimate the prevalence of depressive symptoms, anxiety symptoms and sleeping disturbances in the Italian population during lockdown. The factors that might influence such outcomes were explored. A national cross-sectional survey was performed during the last 14 days of the Italian lockdown. Questionnaires assessed socio-demographics characteristic, behaviors and healthcare access. The outcomes were assessed using Patient Health Questionnaire-2 and Generalized Anxiety Disorder-2. Participants with sleep disturbances completed the Insomnia Severity Index. The sample size was 1515. Depression and anxiety symptom prevalence was 24.7% and 23.2%; 42.2% had sleep disturbances and, among them, 17.4% reported moderate/severe insomnia. Being female, an increased time spent on the internet and an avoidance of activities through peer pressure increased the likelihood of at least one mental health outcome. Increasing age, an absence of work-related troubles and being married or being a cohabitant reduced such a probability. Females and participants with chronic conditions were associated with a higher prevalence of sleep disturbances. It is crucial to study effective interventions, specifically planning strategies, for more vulnerable groups and to consider the role of the internet.

## 1. Introduction

The coronavirus disease 2019 (Covid-19) has already been recognized as a cause of direct and indirect psychological and social consequences that might impact on mental health (MH) not only during the pandemic per se but also in the future [1]. Indeed, the quarantine effects have already been explored during past outbreaks, such as during the outbreak of Severe Acute Respiratory Syndrome (SARS) in 2003 and Ebola in 2014, indicating that the MH impact can be broad, massive and long-lasting [2]. Among the consequences of quarantine, there are acute stress disorders, anxiety, irritability, poor concentration and indecisiveness, deteriorating work performance, post-traumatic stress disorders, high psychological distress, depressive symptoms and insomnia [2]. Data on the pre-existing factors that might predict MH outcomes are conflicting [2], e.g., age, education, gender and having children have been considered both with [3] and without [4] an association with psychological issues. Moreover, the main MH stressors during the quarantine have resulted to be the duration of the quarantine, fears of infection, frustration and boredom, inadequate supplies and inadequate information [2]. However, other authors have outlined how past confinement studies are poorly comparable to this pandemic confinement as the current worldwide long-term home quarantine of masses of individuals, with access to digital means to preserve communication, work and education, is unprecedented [5]. Therefore, there is a strong need for studies on the current situation to define the extent of the impact of COVID-19 and to understand determinants to implement appropriate interventions.

To date, the psychological response during the COVID-19 quarantine has been studied more extensively in China, where several studies have already been carried out [6,7,8,9,10,11]. Findings from China have reported a prevalence of depression during quarantine up to 37% [6], and a prevalence of anxiety up to 35% [7]. In particular, a comparison study found significant differences in the prevalence of depression and anxiety between people in quarantine (22.4% and 12.9%, respectively) and people not in quarantine (11.9% and 6.7%) [8]. Considering factors that might predict mental health, these studies showed conflicting results, for instance on gender, as reported in research conducted in previous epidemics [2]. Indeed, gender seemed to have a significant relationship with mental health outcomes in some works [9,10], while in others this association was not significant [6,7]. Furthermore, other groups of people were found to be more vulnerable and to experience greater mental health issues, such as youths [6,7,11] and people who faced financial stress [8].

In Europe, Italy was the first country to enter a nationwide lockdown [12]. First, lockdown concerned eleven municipalities in Northern Italy beginning from the 23rd February [13], with restrictive measures involving six regions of Northern Italy two days later [14]. Then, more restrictive decrees gradually followed up to the 9th March, when the lockdown measures were extended to the whole Italian territory [12], and 11th March, when tightened restrictions were announced [15]. The initial date for the end of the lockdown was the 3rd April [12], however it was extended step by step up to the 3rd May [16]. During this period, only essential activities were permitted, and only essential shops were allowed to be open and individuals had permission to leave their homes only for demonstrated necessities, such as for health reasons, shopping for basic needs and for work (if working from home was not possible) [15]. On the 3rd May, the total number of confirmed cases of COVID-19 in Italy were 210,717, with 28,884 deaths [17].

Given the above, the present study aimed to estimate the psychological impact of COVID-19 and related restrictive measures through a nationwide cross-sectional survey that evaluated the prevalence of depressive symptoms, anxiety symptoms and sleeping issues in the Italian general population during the last weeks of lockdown. Our main hypothesis was that the impact on mental health may be consistent and comparable across all countries that had to face a lockdown. Additionally, another purpose was to explore through regression models the predictors and determinants that might influence such MH outcomes in this unique context. The objective of these analyses was to identify potentially vulnerable groups or possible modifiable factors in order to have a basis to plan specific and targeted strategies to reduce the burden of mental health issues due to COVID-19 quarantine.

## 2. Materials and Methods

A national cross-sectional study was performed during the last 14 days of the Italian lockdown (between April 19th and May 3rd 2020) through an online questionnaire, distributed through social networks from the institutional pages of the Department of Public Health Sciences (University of Torino). All subjects gave their informed consent for inclusion before they participated in the study. The study was conducted in accordance with the Declaration of Helsinki, and the protocol was approved by the Internal Review Board of the Department of Public Health Sciences (University of Torino). The collected answers were excluded from the final sample if the subjects met one of the exclusion criteria (being underage or living abroad during lockdown). Participation was voluntary and without compensation. The present work is a part of the Covid Collateral Impacts (COCOS) project and is focused on the MH issues of the subjects involved.

The self-administered questionnaire was composed of forty-nine items. A first section investigated the socio-demographic characteristics of the subjects: age, gender, nationality, marital status, educational level, occupation, fear of losing job, economic losses and history of chronic disease were assessed. Some independent variables were coded from the above-mentioned items. Education level was grouped considering the presence of university degree as a binary outcome. The covariate “Activity during lockdown” was recoded, grouping “I do not work” and “My activity is not changed” as “No variation”; “Layoff”, “Parental Leave” and “Paid Vacation” as “Guaranteed Income”; and “My activity is reduced”, “My activity is stopped” and “I lost my job” as “Activity Stop/Reduction”. Finally, a new covariate was created and labelled “Economical struggle” merging the answers about occupation, fear of losing a job and economic losses. A first group, named “Non worker”, included subjects with no occupation. A second group, named “Worker experiencing trouble”, included subjects with an occupation that declared either to have a fear of losing their job or to have faced economic losses and a third group was labelled “worker non-experiencing trouble” and included subjects with an occupation and who are not afraid of losing their job or having had the experience of economic losses.

A second section assessed the amount of hours spent on the internet, the sources of information used, the number of times a subject went out in a week, whether the subject used online grocery or not, whether the respondents avoided physical activity because of a fear of injuries or peer pressure and the habit of wearing facemasks when going out. To perform further statistical analysis, the variable “facemask” was considered as a binary outcome, labelling “other” as the answers “No, I do not think is useful”, “No, I was not able to find one” and “Yes, sometimes”.

In the third section, depressive symptoms were investigated through the Patient Health Questionnaire-2 (PHQ-2), a two-item instrument for depression screening [18]. Anxiety was measured by the Generalized Anxiety Disorder-2 (GAD-2), a two-item questionnaire for anxiety disorders screening [19]. A score of 3 or above represented a higher probability of major depression and anxiety disorders, respectively [18,19]. Additionally, if the subject declared to suffer from sleep disturbances, the Insomnia Severity Index (ISI) was used. The scale is composed of 7 items, each one with a score ranging from 0 to 4. “No clinically significant insomnia” was identified if the final score was between 0 and 7, “Subthreshold insomnia” if it was between 8 and 14, “Clinical insomnia (moderate severity)” if it ranged from 10 to 21 and “Clinical insomnia (severe)” if it was between 22 and 28 [20].

Finally, a fourth section evaluated Healthcare access (HCA). In particular, the survey assessed self-medication and whether scheduled medical services had been delayed.

Descriptive analyses were performed for all variables. The Shapiro–Wilk test was used to test normal distribution of continuous variables. To determine differences between groups defined by each outcome, chi-square tests (when appropriate, Fisher’s exact tests) and Mann–Whitney U tests (when appropriate, Kruskal–Wallis test) were computed. Univariable and multivariable logistic regressions were conducted to assess the influence of independent variables on each binary outcome (results expressed as Odds Ratios (OR), 95% CI). The covariates included in multivariable models were selected using a two-step selection process. A fixed model was used for covariates with a univariable *p*-value <0.05, and a stepwise backward selection process was used for covariates with a univariable *p*-value < 0.25 [21], and with age and gender as potential confounders. SPSS (v25) was used and a two-tailed *p*-value < 0.05 was considered significant. Missing values were excluded.

## 3. Results

The final sample was made of 1515 questionnaires. In fact, 1556 questionnaire were completed, but 41 questionnaires were excluded because they met the exclusion criteria. A description of the sample is provided in Table 1. The median age was 42 years (IQR = 23) and females accounted for 65.6%. Most of the sample came from Northern Italy (75.5%) and most declared to be married or cohabitants (61.1%). The scales used to screen for MH issues returned a 24.7% prevalence of depression symptoms and a 23.2% prevalence of anxiety disorder. Finally, 42.2% of respondents referred to having suffered from trouble sleeping. Among them, 19.9% resulted to have no clinical insomnia, 62.7% to suffer from subthreshold insomnia, 16.3% to suffer from a moderate clinical insomnia and only 1.1% from a severe clinical insomnia.

As reported in Table 2, age resulted to be significantly lower in the group presenting depressive symptoms (*p* < 0.001). Similarly, significant differences based on the presence of depression were recorded considering marital status (*p* < 0.001), economical struggles (*p* = 0.033) and time spent on the internet (*p* < 0.001). The prevalence of depressive symptoms was lower in the group using the newspaper as source of information (*p* = 0.018) and among those who always wear a protective facemask when going out (*p* = 0.005). On the contrary it was higher among subjects declaring to be afraid to leave the home for their needs (*p* < 0.001) and among subjects who avoided activity either because of the fear of injuries (*p* = 0.001) or because of peer pressure (*p* < 0.001).

Similarly, age resulted to be significantly lower in the group presenting anxiety disorder (*p* < 0.001). Interestingly, gender (*p* < 0.001), marital status (*p* < 0.001), education level (*p* = 0.046) and being a healthcare worker (*p* = 0.019) resulted in an association with the prevalence of anxiety. Additionally, significant differences were recorded considering time spent on the internet, either considering the number of hours per day (*p* = 0.015) or the variation during the lockdown (*p* = 0.002). The use of different sources of information—radio (*p* = 0.031), newspapers (*p* = 0.026) and internet (*p* = 0.041)—resulted to be associated with differences in the prevalence of anxiety disorder. Additionally, a higher prevalence was recorded among those who declared to be afraid to leave their home (*p* < 0.001), those who declared that they avoided activity because of a fear of injuries (*p* = 0.023) or because of peer pressure (*p* < 0.001) and among those who used self-medication (p = 0.026). On the contrary, a lower prevalence was registered among subjects who declared to always wear a facemask when going out compared to the others (*p* = 0.026).

As seen for depression and anxiety, the median age in the group suffering from sleep disturbances resulted to be significantly lower (*p* < 0.001). Additionally, the covariate associated with differences in the presence of sleep disturbances were gender (*p* < 0.001), activity during lockdown (*p* = 0.001), the presence of economical struggles (*p* = 0.010) or domestic animals (p = 0.033). Differences in the prevalence of sleep disturbances were associated with the use of the internet, considering the number of hours per day (*p* = 0.019) or the trend since the beginning of the lockdown (*p* = 0.029). Interestingly, no association was found considering the source of information used. An increased frequency of sleep disturbances was associated with being afraid to leave home (*p* < 0.001), having recourse to self-medication (*p* = 0.002) or with avoiding activities either because of a fear of injuries (*p* < 0.001) or because of peer pressure (*p* < 0.001).

Multivariable logistic regression models were used to investigate possible predictors of poor MH and sleep disturbances (Table 3). In particular, considering the presence of depressive symptoms, being married or being a cohabitant resulted to be a protective factor (Adjusted Odd Ratio, AdjOR= 0.67; 95% Confidence Interval C.I.: 0.48–0.94). Similarly, having a job and not experiencing economical struggle significantly reduced the risk of developing depressive symptoms (AdjOR = 0.56; 95% C.I.: 0.38–0.83). Conversely, spending more time connected since the beginning of the lockdown was associated with an increased risk of depression (AdjOR = 1.64; 95% C.I.: 1.07–2.35), as was avoiding activity because of peer pressure (AdjOR = 2.20; 95% C.I.: 1.57–3.10).

Different results can be seen considering the risk factors for anxiety disorder. In fact, females showed a significantly higher risk of presenting anxiety (AdjOR = 2.06; 95% C.I.: 1.44–2.95) and age resulted to be a weak protective factor (AdjOR = 0.98; 95% C.I.: 0.97–1.00). In addition, only avoiding activity because of peer pressure resulted to be a predictor of anxiety (AdjOR = 1.62; 95% C.I.: 1.14–2.29). Finally, predictors of sleep disturbances were investigated. In this case, an increased risk can be found among females (AdjOR = 1.70; 95% C.I.: 1.27–2.28) and subjects with chronic conditions (AdjOR = 1.67; 95% C.I.: 1.15–2.41). No significant association was found with the other variables.

## 4. Discussion

The prevalence of MH issues in the present study is higher than the prevalence recorded in the Italian population before the lockdown: the latest data by the Italian National Statistical Institute indicated a depressive symptoms prevalence over the last two weeks of 5.4% and a severe anxiety prevalence over the last year of 4.2% [22]. During the first weeks of lockdown, the response of the Italian general population was estimated through the Depression, Anxiety and Stress Scale-21 [23,24]. Moderate to extremely high levels of depression were reported in 32.4% [24] and 21.2% of the population [23], while moderate to extremely high levels of anxiety was reported in 18.7% [24] and 32.6% [23]. Moreover, participants experiencing poor sleep were 40.5% before lockdown and 52.4% during lockdown (Pittsburg Sleep Quality Index) [23]. The differences between our study and these relevant works could be explained by the different timing during the lockdown and the different tools used. Additionally, the sample compositions were considerably different to ours, with 71.7% of females with a mean age of 32.94 years in the first study [24], and 67% of females with a mean age of 23.91 years in the second [23].

Furthermore, several pieces of research into MH outcomes among the general population during the COVID-19 quarantine have been conducted in China [6,7,8,9,10,11] and beyond [25,26]. Overall, our results seem consistent with these studies, which have reported a prevalence of depressive symptoms from 16.5% [9] to 37% [6], a prevalence of anxiety symptoms from 12.9% [8] to 35.1% [7], and a prevalence of sleep disturbances from 18.2% [7] to 52.4% [23] (Table 4).

Concerning the predictors that might influence MH, our findings underlined several factors that were mostly coherent with the existing literature.

First, the multivariable models showed a positive association between being female and both anxiety and sleeping disturbances (no significant association with depressive symptoms). The evidence of the relationship between gender and MH outcomes during quarantine is conflicting [2,6,7,11,24,25]. Several studies indicated that females were more prone to report depression and anxiety [24,25] or insomnia [10], while others reported a non-significant interaction of gender with anxiety and depression [6,7] and sleep quality [7], suggesting that men and women might be equally concerned about this pandemic [6]. Conversely, Wang et al. reported that the male gender was significantly associated with higher scores of anxiety and depressive symptoms [9].

Furthermore, our study reported an increasing age to lower the likelihood of anxiety (of depressive symptoms and sleep disturbances only in univariable analyses), consistent with previous literature. Young adults have been reported to be more likely to present depression [6,7,25,26], anxiety [6,7,24,25] and reduced sleep quality [11]. Ozamiz-Etxebarria and colleagues suggested that one explanation could be the fact that a section of young people can be university students, which usually report high levels of mental health issues [27,28] and might experience additional stress due to the necessity to adapt their university career [9,26]. Indeed, delays in academic activities due to COVID-19 have been correlated with anxiety [29]. Besides, young individuals are usually engaged in short-term employment, this being an additional risk-factor for poor MH outcomes [30]. Lastly, younger people might experience higher anxiety levels because they are likely to reach a greater amount of information through social media, which might influence stress [31].

In this regard, the univariable regressions showed that using the internet as source of information has led to a higher probability of anxiety. It is worth mentioning that the most-used source of information was the internet and three-quarters of participants affirmed that their time spent on internet was increased during lockdown. Interestingly, the multivariable models confirmed an association between an increased time spent on the internet and depression, while the univariable analyses showed relationships between all outcomes and at least one variable related to the time spent on the internet. Notably, during the COVID-19 outbreak, social media has been reported to impact on MH spreading fears and panic, causing anxiety mostly among youths [32], and MH problems have been associated with frequent social media exposure [33].

The media often uses risk-elevating messages that can intensify the anxiety of the population [1,34] and media-related distress might boost behaviors that negatively impact on healthcare systems, with subsequent mental and physical health repercussions [1,35]. In fact, Holmes et al. identified the development of guidelines for the media around pandemic reporting as an MH research priority in the context of this pandemic [1].

Moreover, the media has a role in the development of stigma as SARS demonstrated: media contributed to unwarranted public fear, distrust and intolerance towards “dangerous others” [36]. The COVID-19 pandemic has been, and still is, also a pandemic of hate and diffuse stigmatization, particularly against Asian people [37]. As reported by the latest news, during lockdown, hate and stigmatization have been extended to individuals who left their house, e.g., to runners [38]. Such a climate of hate and hostility might partly explain the association we found between depression and anxiety symptoms, and the avoidance of activity due to the pressure exerted by peers.

Finally, workers not experiencing troubles had a lower likelihood of depressive symptoms, consistent with the relationship between working conditions, financial stress and depression during quarantine that has been described in the literature [8,24]. It is worth noting that 76.5% of subjects reported an income reduction due to the pandemic, which is alarming in view of the evidence of a higher risk of stress after an economic recession [39].

Other significant predictors in our analyses need to be further investigated. For example, the relationship between marital status and depression, which has been reported to be non-significant in other studies [4,9], or the role played by chronic conditions, that were associated with anxiety and depression in several studies [9,24,26]. Lastly, although differences in MH outcomes were associated with the duration of quarantine in previous studies [2], no differences were found between Italian geographical areas, despite the different timing of the restrictive measures [12,14].

The present study had some strengths and limitations. To our knowledge, it was the first study investigating MH outcomes among the Italian general population during last weeks of the COVID-19 lockdown. Moreover, the sample was more representative of the Italian population concerning age (mean age of Italian population: 45.7 years [40]) compared to Italian studies on the first weeks of lockdown [23,24]. Nonetheless, the representativeness was less accurate in consideration of gender, geographical distribution and education level. The main limitations were the opportunistic sampling and the cross-sectional design, which restricts causal interpretations. Another limitation was the self-reported measures rather than clinical diagnoses, however the selected tools were validated and commonly used [18,19,20]. Additionally, considering the online distribution, no data about people who refused to participate were collected and no refusal rate was registered.

## 5. Conclusions

In conclusion, our study showed that the Italian general population reported a high prevalence of MH issues during the last weeks of lockdown. Since the impact on MH is expected to persist beyond this critical situation [1], it is crucial to study the most effective interventions to reduce the burden of psychological and social consequences. Specifically, as outlined by our results, it is essential to plan strategies for more vulnerable groups, e.g., youths, and consider the role of the internet on communication and stigmatization.

## Figures and Tables

**Table 1:** Description of the sample.

Variable	Category	N	%
Age ^†^		42	23
Gender	Male	511	34.4
	Female	973	65.6
Geographical Area	North	987	75.5
	Centre	179	13.7
	South	141	10.8
Marital Status	Single/Divorced	577	38.9
	Married/Cohabitant	908	61.1
Education Level	None	1	0.1
	Elementary School	3	0.2
	Middle School	72	4.8
	High School	389	26
	University	1029	68.9
Employment	Unemployed	94	6.2
	Student	108	7.1
	Employed (public sector)	376	24.9
	Employed (private sector)	446	29.5
	Self-employed	208	13.7
	Entrepreneur	37	2.4
	Retiree	224	14.8
	Housewife	20	1.3
Fear of Losing Employment	No	543	85.4
	Yes	93	14.6
Income Reduction	No	46	23.5
	Yes	150	76.5
Activity During Lockdown	I do not work	310	20.7
	My activity is not changed	230	15.3
	Smart working	489	32.6
	Layoff	98	6.5
	Parental Leave	7	0.5
	Paid Vacation	15	1
	My activity is reduced	155	10.3
	My activity is stopped	116	7.7
	I lost my job	18	1.2
	Other	63	4.2
Healthcare Worker	No	1186	79.6
	Yes	304	20.4
Chronic Conditions	No	1171	78.2
	Yes	326	21.8
Domestic Animal	No	944	62.9
	Yes	556	37.1
Shopping Online	No	619	41.6
	Yes	869	58.4
Time Spent on Internet†	Hours/day	9	6
Time Spent on Internet	Stable	322	21.6
	Increased	1119	75.1
	Decreased	22	1.5
	I do not know	27	1.8
Source of Information (TV)	No	454	30
	Yes	1061	70
Source of Information (Radio)	No	1169	77.2
	Yes	346	22.8
Source of Information (Internet)	No	254	16.8
	Yes	1261	83.2
Source of Information (Newspaper)	No	715	47.2
	Yes	800	52.8
Source of Information (Friends)	No	1266	83.6
	Yes	249	16.4
Times Went Out †	Number/Week	3	6
Afraid to Leave Home	No	1019	69.7
	Yes	444	30.3
Do You Wear a Facemask Going Out?	No, I do not think is useful	67	4.4
	No, I was not able to find one	26	1.7
	Yes, sometimes	266	17.7
	Yes, always	1071	71.1
	I do not go out	76	5
Avoidance of Activity (fear of injuries)	No	1145	76.7
	Yes	348	23.3
	Yes	388	26.1
Avoidance of Health Services	No	1299	86.9
	Yes	195	13.1
Self-Medication	No	1420	95
	Yes	74	5

^†^ Continuous variable described as Median and Interquartile Range (IQR).

**Table 2:** Description of the sample stratified according to Depression, Anxiety and Sleep Disturbances.

Variable		Depression (PHQ-2)			Anxiety (GAD-2)			Sleep Disturbances		
		No N (%)	Yes N (%)	*p*-Value	No N (%)	Yes N (%)	*p*-Value	No N (%)	Yes N (%)	*p*-Value
Total		1119 (75.3)	367 (24.7)		1144 (76.8)	345 (23.2)		854 (57.8)	624 (42.2)	
Age ^†^		43 (24)	40 (23)	<0.001 *	44 (26)	37 (17)	<0.001 *	44 (26)	40 (21)	<0.001 *
Gender	Male	389 (77.6)	112 (22.4)	0.132	420 (83.7)	82 (16.3)	<0.001 *	327 (65.5)	172 (34.5)	<0.001 *
	Female	708 (74.1)	248 (25.9)		700 (73.2)	256 (26.8)		509 (53.6)	440 (46.4)	
Geographical Area	North	727 (74.7)	246 (25.3)	0.106	731 (74.8)	246 (25.2)	0.139	558 (57.2)	418 (42.8)	0.242
	Centre	140 (80.5)	34 (19.5)		142 (81.6)	32 (18.4)		107 (61.8)	66 (38.2)	
	South	94 (70.1)	40 (29.9)		99 (73.9)	35 (26.1)		69 (52.3)	63 (47.7)	
Marital Status	Single/Divorced	396 (69.5)	174 (30.5)	<0.001 *	410 (71.8)	161 (28.2)	<0.001 *	313 (55.3)	253 (44.7)	0.164
	Married/Cohabitant	702 (79.1)	185 (20.9)		709 (79.8)	180 (20.2)		521 (59)	362 (41)	
Education Level	High school or lower	335 (73.8)	119 (26.2)	0.289	365 (80)	91 (20)	0.046 *	263 (58.1)	190 (41.9)	0.862
	University	772 (76.4)	239 (23.6)		762 (75.3)	250 (24.7)		578 (57.6)	426 (42.4)	
Activity During Lockdown	No variation	403 (75.9)	128 (24.1)	0.114	418 (78.7)	113 (21.3)	0.197	330 (62.6)	197 (37.4)	0.001 *
	Smart working	377 (78.5)	103 (21.5)		377 (78.1)	106 (21.9)		282 (58.6)	199 (41.4)	
	Guaranteed income	86 (72.9)	32 (27.1)		85 (72)	33 (28)		55 (47.4)	61 (52.6)	
	Activity Stop/Reduction	201 (71)	82 (29)		209 (73.6)	75 (26.4)		143 (50.9)	138 (49.1)	
Economical Struggle	Non worker	317 (72.5)	120 (27.5)	0.033 *	345 (78.9)	92 (21.1)	0.153	261 (60.3)	172 (39.7)	0.010 *
	Worker experiencing trouble	175 (73.2)	64 (26.8)		173 (72.4)	66 (27.6)		118 (49.6)	120 (50.4)	
	Worker nonexperiencing trouble	455 (79.1)	120 (20.9)		445 (77)	133 (23)		349 (60.4)	229 (39.6)	
Healthcare Worker	No	874 (75.1)	290 (24.9)	0.953	910 (78)	257 (22)	0.019 *	672 (57.8)	490 (42.2)	0.727
	Yes	225 (75.3)	74 (24.7)		214 (71.6)	85 (28.4)		169 (66.7)	129 (43.3)	
Chronic Conditions	No	866 (75)	289 (25)	0.766	875 (75.8)	280 (24.2)	0.080	676 (58.7)	475 (41.3)	0.119
	Yes	238 (75.8)	76 (24.2)		255 (80.4)	62 (19.6)		169 (53.8)	145 (46.2)	
Domestic Animal	No	697 (75.2)	230 (24.8)	0.986	728 (78.4)	201 (21.6)	0.055	555 (59.9)	371 (40.1)	0.033 *
	Yes	410 (75.2)	135 (24.8)		404 (74)	142 (26)		294 (54.2)	248 (45.8)	
Shopping Online	No	461 (75.8)	147 (24.2)	0.618	480 (78.9)	128 (21.1)	0.077	357 (59.1)	247 (40.9)	0.273
	Yes	637 (74.7)	216 (25.3)		641 (75)	214 (25)		479 (56.2)	373 (43.8)	
Time Spent on Internet (Amount) †	Hours/day	9 (6)	9 (6)	0.214	9 (6)	9 (6)	0.015 *	8.5 (7)	9 (6)	0.019 *
Time Spent on Internet (Trend)	Stable	270 (82.8)	56 (17.2)	<0.001 *	265 (81)	62 (19)	0.002 *	203 (63)	119 (37)	0.029 *
	Increased	808 (73.4)	293 (26.6)		835 (75.8)	267 (24.2)		613 (55.9)	483 (44.1)	
	Decreased	11 (52.4)	10 (47.6)		10 (47.6)	11 (52.4)		10 (47.6)	11 (52.4)	
	I don’t know	21 (77.8)	6 (22.2)		23 (85.2)	4 (14.8)		20 (74.1)	7 (25.9)	
Source of Information (TV)	No	328 (73.5)	118 (26.5)	0.303	330 (73.8)	117 (26.2)	0.072	252 (56.5)	194 (43.5)	0.513
	Yes	791 (76.1)	249 (23.9)		814 (78.1)	228 (21.9)		602 (58.3)	430 (41.7)	
Source of Information (Radio)	No	852 (74.4)	293 (25.6)	0.144	868 (75.5)	281 (24.5)	0.031 *	657 (57.5)	486 (42.5)	0.666
	Yes	267 (78.3)	74 (21.7)		276 (81.2)	64 (18.8)		197 (58.8)	138 (41.2)	
Source of Information (Internet)	No	180 (74.7)	61 (25.3)	0.809	199 (81.9)	44 (18.1)	0.041 *	146 (60.3)	96 (39.7)	0.380
	Yes	939 (75.4)	306 (24.6)		945 (75.8)	301 (24.2)		708 (57.3)	528 (42.7)	
Source of Information (Newspaper)	No	506 (72.5)	192 (27.5)	0.018 *	519 (74.2)	180 (25.8)	0.026 *	382 (55.2)	310 (44.8)	0.060
	Yes	613 (77.8)	175 (22.2)		625 (79.1)	165 (20.9)		472 (60.1)	314 (39.9)	
Source of Information (Friends)	No	945 (76.1)	296 (23.9)	0.089	962 (77.3)	282 (22.7)	0.302	719 (58.2)	517 (41.8)	0.492
	Yes	174 (71)	71 (29)		182 (74.3)	63 (25.7)		135 (55.8)	107 (44.2)	
Times Went Out †	Number/Week	3 (6)	3 (6)	0.560	3 (6)	4 (7)	0.011 *	3 (6)	3 (6)	0.943
Afraid to Leave Home	No	790 (78.7)	214 (21.3)	<0.001 *	808 (80.3)	198 (19.7)	<0.001 *	619 (61.9)	381 (38.1)	<0.001 *
	Yes	294 (67)	145 (33)		299 (68.1)	140 (31.9)		210 (47.9)	228 (52.1)	
Facemask	Other	303 (70.5)	127 (29.5)	0.005 *	314 (73)	116 (27)	0.026 *	240 (55.9)	189 (44.1)	0.373
	Yes, always	815 (77.3)	239 (22.7)		827 (78.4)	228 (21.6)		611 (58.5)	434 (41.5)	
Avoidance of Activity (Fear of Injuries)	No	871 (77.2)	257 (22.8)	0.001 *	881 (78)	248 (22)	0.023 *	675 (60.2)	447 (39.8)	<0.001 *
	Yes	236 (68.6)	108 (31.4)		248 (72.1)	96 (27.9)		166 (48.5)	176 (51.5)	
Avoidance of Activity (Peer Pressure)	No	864 (79.6)	221 (20.4)	<0.001 *	875 (80.6)	210 (19.4)	<0.001 *	661 (61.3)	417 (38.7)	<0.001 *
	Yes	239 (62.7)	142 (37.3)		248 (64.9)	134 (35.1)		179 (47.1)	201 (52.9)	
Avoidance of Health Services	No	980 (75.9)	311 (24.1)	0.162	1000 (77.3)	294 (22.7)	0.289	750 (58.5)	533 (41.5)	0.177
	Yes	139 (71.3)	56 (28.7)		144 (73.8)	51 (26.2)		104 (53.3)	91 (46.7)	
Self-Medication	No	1068 (75.6)	344 (24.4)	0.191	1095 (77.4)	320 (22.6)	0.026 *	824 (58.7)	580 (41.3)	0.002 *
	Yes	51 (68.9)	23 (31.1)		49 (66.2)	25 (33.8)		30 (40.5)	44 (59.5)	

^†^ Continuous Variable. Described as Median and Interquartile Range (IQR). * Two tailed *p*-value <0.05 (significant).

**Table 3:** Predictors of depression, anxiety and sleep disturbances (AdjOR: Adjusted Odds Ratio; CI: Confidence Interval).

Variable		Sleep Disturbances		
		AdjOR (95% CI)	AdjOR (95% CI)	AdjOR (95% CI)
Age		1 (0.99–1.02)	0.98 (0.97–1.00) *	0.99 (0.98–1.00)
Gender	Male	-	-	-
	Female	1.20 (0.86–1.68)	2.06 (1.44–2.95) *	1.70 (1.27–2.28) *
Marital Status	Single/Divorced	-	-	-
	Married/Cohabitant	0.67 (0.48–0.94) *	0.73 (0.52–1.03)	-
Education Level	High school or lower	-	-	-
	University	-	1.20 (0.83–1.73)	-
Activity During Lockdown	No variation	-	-	-
	Smart working	-	-	1.13 (0.75–1.69)
	Guaranteed income	-	-	1.55 (0.84–2.84)
	Activity Stop/Reduction	-	-	1.23 (0.78–1.94)
Economical Struggle	Non worker	-	-	-
	Worker experiencing trouble	0.75 (0.48–1.17)	-	1.18 (0.73–1.89)
	Worker nonexperiencing trouble	0.56 (0.38–0.83) *	-	0.84 (0.58–1.24)
Chronic Conditions	No	-	-	-
	Yes	-	-	1.67 (1.15–2.41) *
Domestic Animal	No	-	-	-
	Yes	-	-	1.18 (0.88–1.58)
Shopping Online	No	-	-	-
	Yes	-	-	-
Time Spent on Internet (Amount)	Hours/day	1.04 (1–1.09)	1.02 (0.97–1.06)	1.04 (1.00–1.08)
Time Spent on Internet (Trend)	Stable	-	-	-
	Increased	1.64 (1.07–2.53) *	1.09 (0.72–1.66)	1.07 (0.76–1.52)
	Decreased	3.02 (0.78–11.65)	3.33 (0.85–13.06)	0.87 (0.25–3.01)
	I don’t know	0.39 (0.05–3.19)	0.69 (0.14–3.43)	0.46 (0.12–1.76)
Source of Information (Radio)	No	-	-	-
	Yes	0.69 (0.46–1.06)	0.82 (0.54–1.24)	-
Source of Information (Internet)	No	-	-	-
	Yes	-	1.02 (0.62–1.68)	-
Source of Information (Newspaper)	No	-	-	-
	Yes	0.88 (0.64–1.22)	0.84 (0.61–1.17)	-
Afraid to Leave Home	No	-	-	-
	Yes	1.33 (0.93–1.90)	1.58 (1.10–2.27)	1.30 (0.94–1.79)
Facemask	Other	-	-	-
	Yes, always	0.74 (0.53–1.04)	0.83 (0.59–1.18)	-
Avoidance of Activity (Fear of Injuries)	No	-	-	-
	Yes	1.19 (0.81–1.75)	1.29 (0.87–1.91)	1.38 (0.98–1.94)
Avoidance of Activity (Peer Pressure)	No	-	-	-
	Yes	2.20 (1.57–3.10) *	1.62 (1.14–2.29) *	1.35 (0.99–1.85)
Self-Medication	No	-	-	-
	Yes	-	1.89 (0.97–2.68)	1.46 (0.77–2.76)

* Two tailed *p*-value < 0.05 (significant).

**Table 4:** Prevalence of depression, anxiety and sleep disturbances. Comparison of the present study with other relevant works.

Relevant Works	Country	Sample Size	Depression		Anxiety		Sleep Disturbances	
			Test	Frequency (%)	Test	Frequency (%)	Test	Frequency (%)
Present work	Italy	1515	PHQ-2	24.7	GAD-2	23.2	-	42.2
Cellini 2020 [23]	Italy	1310	DASS-21	21.2	DASS-21	32.6	PSQI	52.4
Mazza 2020 [24]	Italy	2766	DASS-21	32.4	DASS-21	18.7	-	-
Ahmed 2020 [6]	China	1074	BDI	37.1	BAI	29	-	-
Huang 2020 [7]	China	7236	CES-D	20.1	GAD-7	35.1	PSQI	18.2
Lei 2020 [8]	China	1593	SDS	22.4	SAS	12.9	-	-
Wang 2020 [9]	China	1304	DASS-21	16.5	DASS-21	28.8	-	-
Gonzàlez-Sanguino 2020 [25]	Spain	3480	PHQ-2	18.7	GAD-2	21.6	-	-

PHQ-2 Patient Health Questionnaire-2; GAD-2: Generalized Anxiety Disorder-2; DASS-21: Depression, Anxiety and Stress Scale-21; PSQI: Pittsburg Sleep Quality Index; BDI: Beck Depression Inventory; BAI: Beck Anxiety Inventory; CES-D: Center for Epidemiology Scale for Depression; GAD-7: Generalized Anxiety Disorder-7: SDS: self-rating depression scale; SAS: Self-rating anxiety scale.
